# Supplementary material for: Providing an e-cigarette starter kit for smoking cessation and reduction as adjunct to usual care to smokers with a mental health condition: findings from the ESCAPE feasibility study
Source: BMC Psychiatry. 2025 Jan 3;25:13. doi: 10.1186/s12888-024-06387-7 (PMC11699696; doi:10.1186/s12888-024-06387-7)
Supplement: Supplementary file 1 — Supplementary Material 1 [file 12888_2024_6387_MOESM1_ESM.docx]

**Supplementary material**

Supplementary Table 1. Interview topic guide.

|  | **MAIN QUESTION** | **PROMPTS/FOLLOW-UP** |
| --- | --- | --- |
| OPENING QUESTIONS: | Tell us a little about yourself. | Age, where abouts in the UK they live, if they work etc. |
| OPENING QUESTIONS, SMOKING HISTORY: | Ask the participant about their smoking history. | when and why they started etc. |
| 1. SMOKING RELATED OPENING QUESTIONS | 1. Have your smoking habits changed since taking part in the study? | -If so how? If not why not?”  -Why participant relapsed? |
|  | 1. Have you previously tried to quit smoking? | -If yes, please can you describe what methods you used?  -How did this attempt using e-cigarettes compare?  -if no previous attempt, how was this time different? |
| 1. INTERVENTION CONTENT AND DELIVERY (*for participants to the intervention group only*) | 1. Overall, was being offered an e-cigarette helpful? | -did they use it?  Why? Positives? Negatives? |
|  | 1. Did you understand how to use the e-cigarette and obtain additional liquid? | -Prompts: explanation from clinician; time taken, leaflet content, how did they get most if their info, clinician/leaflet both. How could the format of the information be improved. |
|  | 1. Did you find the leaflet useful? | -Why? Was it revisited – in what circumstances |
|  | 1. In your opinion was the clinician the best person to offer the e-cigarette and advice? | -Why? How could this be improved? |
|  | 1. Effects on usual care | -Was there an impact on your appointment and care received? What would help remove this impact? |
| 1. INTERVENTION EFFECTS (for participants to the intervention group only) | 1. What benefits did you get from using the e-cigarette if you used it. | -Prompts: health, social, financial…  Were there any negative effects? |
|  | 1. Which flavours and strengths worked best for you | Why? any flavours they would prefer? |
|  | 1. Would you recommend this type of support for other people with a mental illness | -If so, why not? If not, why not?  -Anything in particular that would increase or reduce likelihood of recommending |
| 1. RECRUITMENT & CONSENT | 1. Thinking back to when you decided to take part, were there any aspects that you think could be improved? | -Prompt – letter, follow-up text or phone call, sufficient information provided, contact with the research team, ease of communication, and choices offered. |
| 1. DATA COLLECTION | 1. Which method did you choose to complete the questionnaires | -Why did you choose that method? |
|  | 1. Did you require any support to complete the questionnaire | -If so what? |
|  | 1. Please tell us how acceptable the questionnaire was to you? | -Prompt - length, did the questions seem appropriate |
|  | 1. If you were to need to complete the questionnaire again, is there any way that it could be improved |  |
| 1. CLOSING QUESTIONS | 1. In general, how was your experience of participating in this research study? | -Prompts – previous research experience, what could be improved, what worked |
|  | 1. Why did you decide to take part in this study? |  |
|  | 1. Is there anything else you would like to add about participating in this study? |  |
| Thank the participant for their time. | | |

Supplementary Table 2: Group differences with scaled confidence intervals (complete case analysis)

| **Measure** | **CO validated sustained abstinence** | **Point prevalence (24h) abstinence** | **Smoking reduction** |
| --- | --- | --- | --- |
| 95% CI | -18.7%, 41.6% | -2.1%, 58.9% | 24.3%, 80.4% |
| 90% CI | -7.1%, 33.3% | 3.6%, 53.4% |  |
| 80% CI | -0.9%, 28.2% |  |  |

Supplementary Table 3: Changes in GAD-7 and PHQ-9 items between baseline and follow-up

|  | Control group M (SD) | Intervention group M (SD) | 80% CI for mean difference |
| --- | --- | --- | --- |
| **GAD-7 items** |  |  |  |
| 1. Feeling nervous, anxious or on edge | 0.3 (1.7) | 0.3 (1.1) | -0.9, 0.5 |
| 2. Not being able to stop or control worrying | 0.6 (1.6) | 0.9 (1.2) | -0.9, 0.5 |
| 3. Worrying too much about different things | 0.4 (1.7) | 0.8 (1.6) | -1.2, 0.5 |
| 4. Trouble relaxing | 0.2 (1.5) | 0.5 (1.4) | -1.0, 0.4 |
| 5. Being so restless that it is hard to sit still | 0.6 (1.2) | 0.5 (1.5) | -0.6, 0.7 |
| 6. Becoming easily annoyed or irritable | 0.3 (0.6) | 0.2 (1.3) | -0.4, 0.5 |
| 7. Feeling afraid as if something awful might happen | 0.8 (1.3) | 0.4 (1.1) | -0.2, 1.1 |
| GAD-7 overall score | 3.2 (8.3) | 3.5 (6.6) | -4.2, 3.6 |
| **PHQ-9 items** |  |  |  |
| 1. Little interest or pleasure in doing things | 0.3 (0.9) | 0.4 (0.9) | -0.6, 0.3 |
| 2. Feeling down, depressed, or hopeless | 0.6 (0.9) | 0.3 (1.0) | -0.2, 0.8 |
| 3. Trouble falling or staying asleep, or sleeping too much | -0.3 (1.2) | 0.3 (1.1) | -1.1, 0.1 |
| 4. Feeling tired or having little energy | 0.3 (1.2) | 0.3 (1.4) | -0.6, 0.7 |
| 5. Poor appetite or overeating | 0.4 (1.6) | 0.7 (1.5) | -1.0, 0.5 |
| 6. Feeling bad about yourself – or that you are a failure or have let yourself or your family down | 0.6 (1.8) | 0.3 (1.2) | -0.6, 1.1 |
| 7. Trouble concentrating on things, such as reading the newspaper or watching television | 0.3 (1.5) | 0.0 (0.8) | -0.4, 0.9 |
| 8. Moving or speaking so slowly that other people could have noticed. Or the opposite. | 0.3 (1.2) | 0.3 (1.4) | -0.6, 0.7 |
| 9. Thoughts that you would be better off dead, or hurting yourself in some way | 0.2 (0.4) | -0.2 (0.9) | 0.1, 0.7 |
| PHQ-9 overall score | 2.2 (8.1) | 1.6 (6.5) | -3.3, 4.4 |

M=Mean, SD=Standard Deviation, CI=Confidence Intervals

Supplementary Table 4**:** General mood and physical symptoms and adverse events as function of group assignment

|  | **Control group M (SD)** | **Intervention group M (SD)** | **80% CI for mean difference** |
| --- | --- | --- | --- |
| **Mood and Physical symptoms** | | | |
| Mouth sore | 1.1 (0.3) | 1.2 (0.8) | -0.4, 0.2 |
| Constipation | 1.3 (0.9) | 1.9 (1.1) | -1.0, 0.0 |
| Cough | 1.3 (0.7) | 1.3 (0.6) | -0.3, 0.3 |
| Depression | 2.2 (1.3) | 2.7 (1.7) | -1.3, 0.2 |
| Irritable | 2.0 (0.9) | 2.9 (1.3) | -1.4, -0.3 |
| Anxious | 2.3 (1.1) | 2.5 (1.4) | -0.9, 0.3 |
| Restless | 1.6 (1.0) | 2.3 (1.6) | -1.4, -0.1 |
| Hungry | 1.5 (1.0) | 2.0 (1.1) | -1.0, 0.0 |
| Poor concentration | 2.3 (1.1) | 2.7 (1.4) | -1.1, 0.2 |
| 3Poor sleep | -1.9 (1.1) | 2.4 (1.6) | -1.2, 0.2 |
|  | **% (n)** | **% (n)** | **80% CI for % difference** |
| **Adverse events** | | | |
| Nausea | 9.1 (2) | 23.8 (5) | 0.8, 29.2 |
| Throat/mouth irritation | 9.1 (2) | 14.3 (3) | -7.9, 18.6 |
| Irritation | 18.2 (4) | 38.1 (8) | 2.3, 36.1 |
| Depression | 22.7 (5) | 42.9 (9) | 1.8, 36.8 |
| Restlessness | 9.1 (2) | 28.6 (8) | 12.6, 44.0 |
| Poor concentration | 27.3 (6) | 38.1 (8) | -7.4, 28.2 |
| Increased appetite | 4.6 (1) | 23.8 (5) | 5.7, 33.2 |
| Light headedness | 9.1 (2) | 14.3 (3) | -7.9, 18.6 |
| Disturbed sleep | 18.2 (4) | 28.6 (6) | -6.2, 26.5 |
| Dry mouth and throat | 9.1 (2) | 23.8 (5) | 0.8, 29.2 |
| Shortness of breath | 18.2 (4) | 19.1 (4) | -14.4, 16.3 |
| Headache | 18.2 (4) | 19.1 (4) | -14.4, 16.3 |
| Wheezing | 0.0 (0) | 9.5 (2) | 0.7, 20.9 |
| Cough | 18.2 (4) | 14.3 (3) | -18.4, 10.9 |
| Phlegm | 22.7 (5) | 14.3 (3) | -23.4, 7.0 |
| Other | 0.0 (0) | 4.8 (1) | -3.0, 14.7 |

Answer options 5-point Likert scale, range 1=not at all to 5= extremely, M=mean, SD=Standard Deviation, CI=Confidence Interval

Supplementary Table 5. Breakdown of intervention cost

| Cost component | Cost per participant |
| --- | --- |
| Intervention group |  |
| Training |  |
| Preparation of training materials | £17 |
| Staff cost (trainer) for face-to-face sessions | £29 |
| Staff cost (trainees) for both face-to-face training and NCSTS online training | £82 |
| Consumables | £1 |
| Total training cost | £129 |
|  |  |
| Brief consultation | £3 |
| Bespoke information leaflet | £8 |
| e-cigarette |  |
| Device | £11 |
| e-liquid | £9 |
| Total intervention cost (without training) | £31 |
| Total intervention cost (with training) | £160 |
|  |  |
| Control group |  |
| Pharmacotherapies for smoking cessation | £4 |
| Primary care related to smoking cessation | £20 |
| Total smoking cessation cost | £24 |

Supplementary Table 6. Recruitment issues and mitigation strategies

| **Issue** | **Mitigation** |
| --- | --- |
| 1. Some Trust sites proved unsuitable to support the study after the trial commenced (e.g., seeing patients solely in their homes, not on site; not having spaces/rooms for patients and researchers to meet). | Identify and enrol only Trusts/sites which can support the study (as per protocol). |
| 1. Small caseloads per clinician and low numbers of new referrals: recruitment sources were exhausted quickly at some sites. | Determine case load size and average number of new referrals to gauge how many clinicians per Trust should be engaged as a minimum. |
| 1. Primary care: clinician had no availability to book in trial participants for several weeks. | Participating sites should be able to confirm that they can accommodate trial patients within 2-3 weeks. |
| 1. Exclusion criteria relatively restrictive, specifically regarding ‘patients currently also receiving treatment for a substance abuse disorder’. | Consider revising criterion to include patients receiving treatment for drug and alcohol use, unless it is the primary diagnosis. |
| 1. Identification of participating sites was overall fairly opportunistic - better opportunities for recruitment, e.g., via weekly clozapine clinics, were likely missed. | Develop a list of criteria or requirements for Trusts to sign up to before joining the study and identify suitable sites using a checklist. |
| 1. Sites offering comprehensive smoking cessation support as standard care were excluded. | Include sites irrespective of usual care standard, if other requirements to support recruitment are met (this being a pragmatic trial). |
| 1. Best recruiting Trust: 3 participants/month (original target for full RCT: 8/month over 19 months). | Adjust target and increase number of Trusts involved based on an estimated recruitment rate of 3 participants/trust |
| 1. Attrition rate | Add text messages as a mean of follow-up and use non-contingent incentives. |


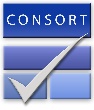
CONSORT checklist of information to include when reporting a pilot or feasibility trial

| Section/Topic | Item No | Checklist item | Reported on page No |
| --- | --- | --- | --- |
| Title and abstract | | | |
|  | 1a | Identification as a pilot or feasibility randomised trial in the title | 1 |
|  | 1b | Structured summary of pilot trial design, methods, results, and conclusions (for specific guidance see CONSORT abstract extension for pilot trials) | 2-3 |
| Introduction | | | |
| Background and objectives | 2a | Scientific background and explanation of rationale for future definitive trial, and reasons for randomised pilot trial | 4-5 |
|  | 2b | Specific objectives or research questions for pilot trial | 5-6 |
| Methods | | | |
| Trial design | 3a | Description of pilot trial design (such as parallel, factorial) including allocation ratio | 6 |
|  | 3b | Important changes to methods after pilot trial commencement (such as eligibility criteria), with reasons | n/a |
| Participants | 4a | Eligibility criteria for participants | 7 |
|  | 4b | Settings and locations where the data were collected | 7 |
|  | 4c | How participants were identified and consented | 6-7 |
| Interventions | 5 | The interventions for each group with sufficient details to allow replication, including how and when they were actually administered | 8-9 |
| Outcomes | 6a | Completely defined pre-specified assessments or measurements to address each pilot trial objective specified in 2b, including how and when they were assessed | 10-13 |
|  | 6b | Any changes to pilot trial assessments or measurements after the pilot trial commenced, with reasons | n/a |
|  | 6c | If applicable, prespecified criteria used to judge whether, or how, to proceed with future definitive trial | 10-13 |
| Sample size | 7a | Rationale for numbers in the pilot trial | 13 |
|  | 7b | When applicable, explanation of any interim analyses and stopping guidelines | n/a |
| Randomisation: |  |  |  |
| Sequence generation | 8a | Method used to generate the random allocation sequence | 7-8 |
|  | 8b | Type of randomisation(s); details of any restriction (such as blocking and block size) | 7-8 |
| Allocation concealment mechanism | 9 | Mechanism used to implement the random allocation sequence (such as sequentially numbered containers), describing any steps taken to conceal the sequence until interventions were assigned | 7-8 |
| Implementation | 10 | Who generated the random allocation sequence, who enrolled participants, and who assigned participants to interventions | 6-7 |
| Blinding | 11a | If done, who was blinded after assignment to interventions (for example, participants, care providers, those assessing outcomes) and how | 8 |
|  | 11b | If relevant, description of the similarity of interventions | 7-8 |
| Statistical methods | 12 | Methods used to address each pilot trial objective whether qualitative or quantitative | 12-13 |
| Results | | | |
| Participant flow (a diagram is strongly recommended) | 13a | For each group, the numbers of participants who were approached and/or assessed for eligibility, randomly assigned, received intended treatment, and were assesses for each objective | 34. figure 1 |
|  | 13b | For each group, losses and exclusions after randomisation, together with reasons | 16 |
| Recruitment | 14a | Dates defining the periods of recruitment and follow-up | 6, 9 |
|  | 14b | Why the pilot trial ended or was stopped | n/a |
| Baseline data | 15 | A table showing baseline demographic and clinical characteristics for each group | 34, Table 1 |
| Numbers analysed | 16 | For each objective, number of participants (denominator) included in each analysis. If relevant, these numbers should be by randomised group | 15-22 |
| Outcomes and estimation | 17 | For each objective, results including expressions of uncertainty (such as 95% confidence interval) for any estimates. If relevant, these numbers should be by randomised group | 15-22 |
| Ancillary analyses | 18 | Results of any other analyses performed that could be used to inform the future definitive trial | 15-22 |
| Harms | 19 | All important harms or unintended effects in each group (for specific guidance see CONSORT for harms) | 19 |
|  | 19a | If relevant, other important unintended consequences | n/a |
| Discussion | | | |
| Limitations | 20 | Pilot trial limitations, addressing sources of potential bias and remaining uncertainty about feasibility | 25 |
| Generalisability | 21 | Generalisability (applicability) of pilot trial methods and findings to future definitive trial and other studies | 23-25 |
| Interpretation | 22 | Interpretation consistent with pilot trial objectives and findings, balancing potential benefits and harms, and considering other relevant evidence | 22-25 |
|  | 22a | Implications for progression from pilot to future definitive trial, including any proposed amendments | 23-25 |
| Other information | | |  |
| Registration | 23 | Registration number for pilot trial and name of trial registry | 3 |
| Protocol | 24 | Where the pilot trial protocol can be accessed, if available | n/a |
| Funding | 25 | Sources of funding and other support (such as supply of drugs), role of funders | 26 |
|  | 26 | Ethical approval or approval by research review committee, confirmed with reference number | 6, 26 |

*We strongly recommend reading this statement in conjunction with the CONSORT 2010 Explanation and Elaboration for important clarifications on all the items. If relevant, we also recommend reading CONSORT extensions for cluster randomised trials, non-inferiority and equivalence trials, non-pharmacological treatments, herbal interventions, and pragmatic trials. Additional extensions are forthcoming: for those and for up-to-date references relevant to this checklist, see [www.consort-statement.org](http://www.consort-statement.org).

Supplementary Table 7. Behaviour change techniques (BCTs) included in the intervention, coded against a 44-item taxonomy of BCTs used in behavioural smoking cessation interventions.

| **Specific focus on behaviour (B) and addressing motivation (M)** | BCTs present | Instantiation in Intervention arm |
| --- | --- | --- |
| BM1. Provide information on consequences of smoking and smoking cessation | Yes | Leaflet |
| BM2. Boost motivation and self-efficacy |  |  |
| BM3. Provide feedback on current behaviour |  |  |
| BM4. Provide rewards contingent on successfully stopping smoking |  |  |
| BM5. Provide normative information about others’ behaviour and experiences |  |  |
| BM6. Prompt commitment from the client here and then |  |  |
| BM7. Provide rewards contingent on effort or progress |  |  |
| BM8. Strengthen ex-smoker identity |  |  |
| BM9. Identify reasons for wanting and not wanting to stop smoking |  |  |
| BM10. Explain the importance of abrupt cessation |  |  |
| BM11. Measure expired-air carbon monoxide (CO) concentration |  |  |
| **Specific focus on behaviour (B) and maximizing self-regulatory capacity/skills (S)** |  |  |
| BS1. Facilitate barrier identification and problem solving | Yes | Leaflet |
| BS2. Facilitate relapse prevention and coping |  |  |
| BS3. Facilitate action planning/develop treatment plan |  |  |
| BS4. Facilitate goal setting | Yes | Verbal advice |
| BS5. Prompt review of goals |  |  |
| BS6. Prompt self-recording |  |  |
| BS7. Advise on changing routine |  |  |
| BS8. Advise on environmental restructuring |  |  |
| BS9. Set graded tasks |  |  |
| BS10. Advise on conserving mental resources |  |  |
| BS11. Advise on avoiding social cues for smoking |  |  |
| **Promote adjuvant activities (A)** |  |  |
| A1. Advise on stop-smoking medication |  |  |
| A2. Advise on/facilitate use of social support |  |  |
| A3. Adopt appropriate local procedures to enable clients to obtain free medication |  |  |
| A4. Ask about experiences of stop smoking medication that the smoker is using |  |  |
| A5. Give options for additional and later support | Yes | Verbal advice |
| **General aspects of the interaction (R) focusing on the delivery of the intervention (D)** |  |  |
| RD1. Tailor interactions appropriately | Yes | verbal advice |
| RD2. Emphasize choice | Yes | verbal advice /leaflet |
| **General aspects of the interaction (R) focusing on information gathering (I)** |  |  |
| RI1. Assess current and past smoking behaviour | Yes | baseline questionnaire |
| RI2. Assess current readiness and ability to quit | Yes | baseline questionnaire |
| RI3. Assess past history of quit attempts | Yes | baseline questionnaire |
| RI4. Assess withdrawal symptoms |  |  |
| **General aspects of the interaction (R) focusing on general communication (C)** |  |  |
| RC1. Build general rapport |  |  |
| RC2. Elicit and answer questions | Yes | verbal advice |
| RC3. Explain the purpose of CO monitoring |  |  |
| RC4. Explain expectations regarding treatment programme | Yes | verbal advice/leaflet |
| RC5. Offer/direct towards appropriate written materials | Yes | leaflet |
| RC6. Provide information on withdrawal symptoms |  |  |
| RC7. Use reflective listening |  |  |
| RC8. Elicit client views |  |  |
| RC9. Summarize information/confirm client decisions | Yes | verbal advice |
| RC10. Provide reassurance |  |  |

**Intervention leaflet**

**
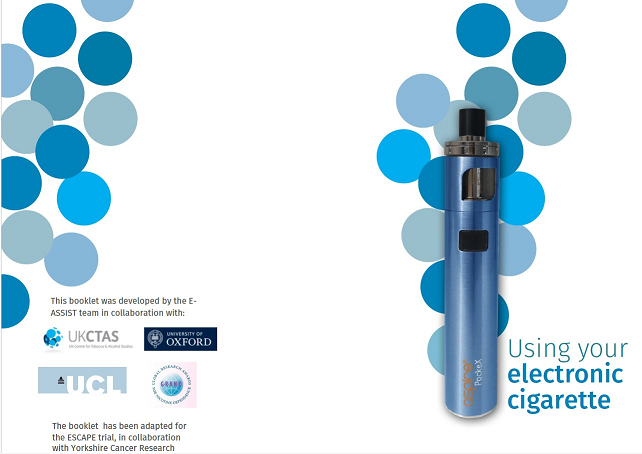
**

**
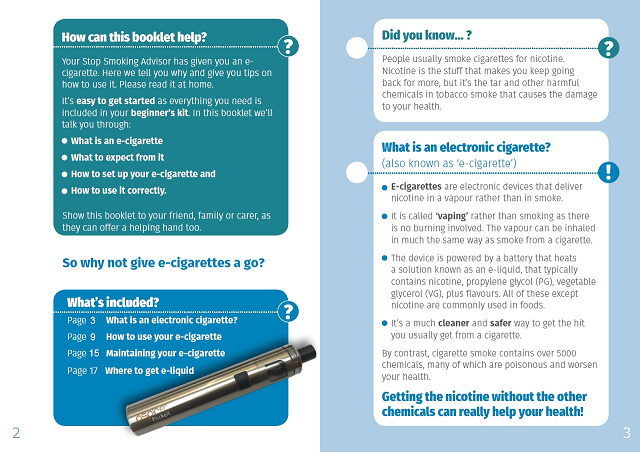
**

**
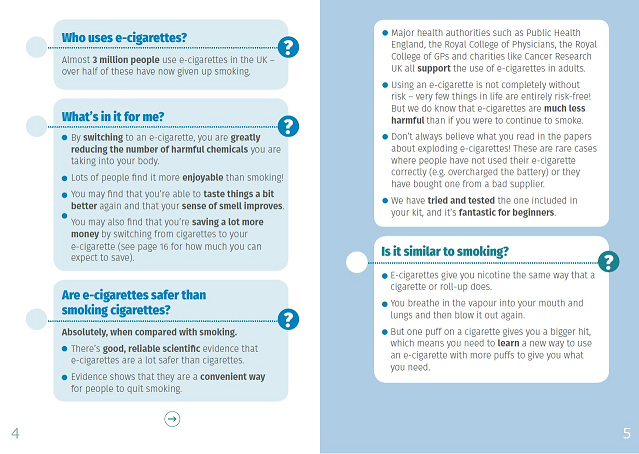
**

**
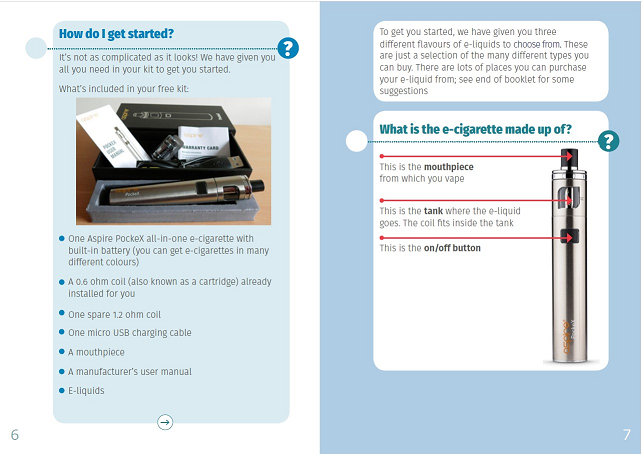
**

**
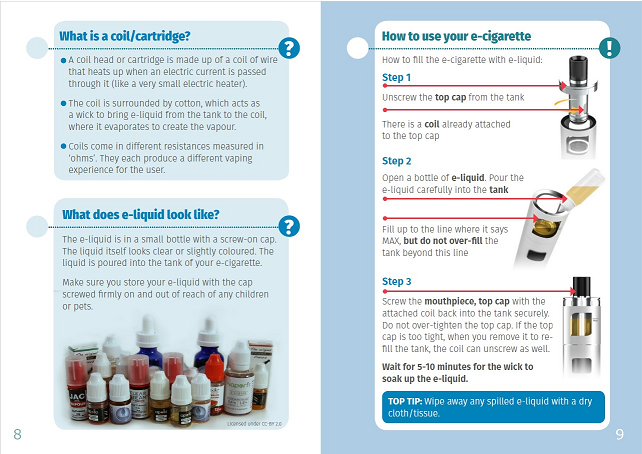
**

**
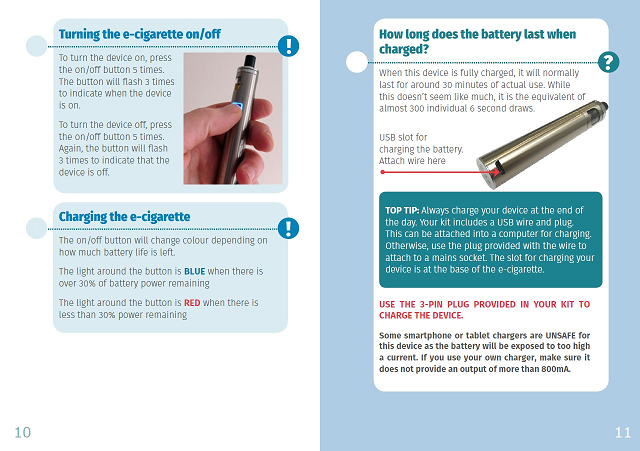
**

**
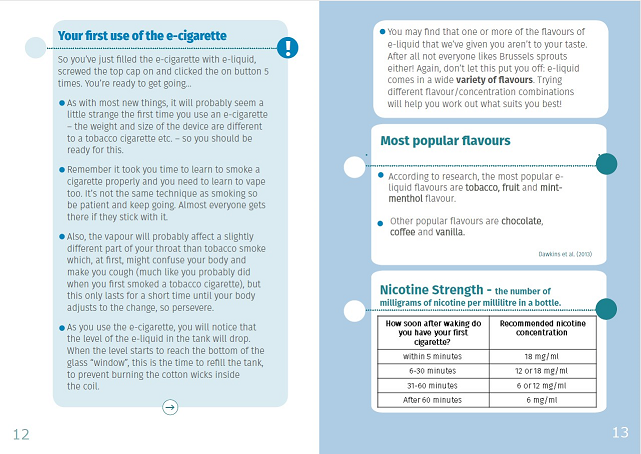
**

**
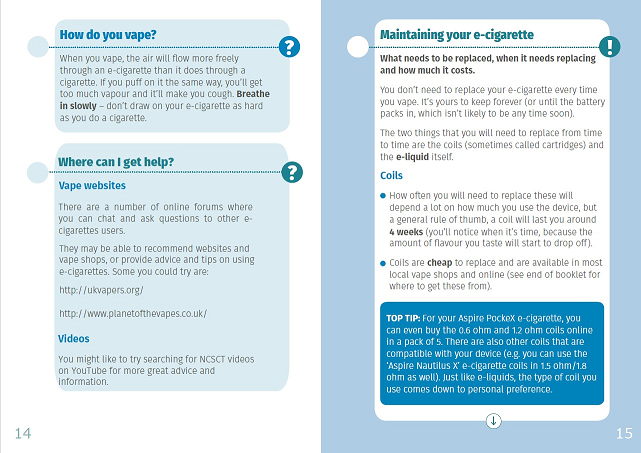
**

**
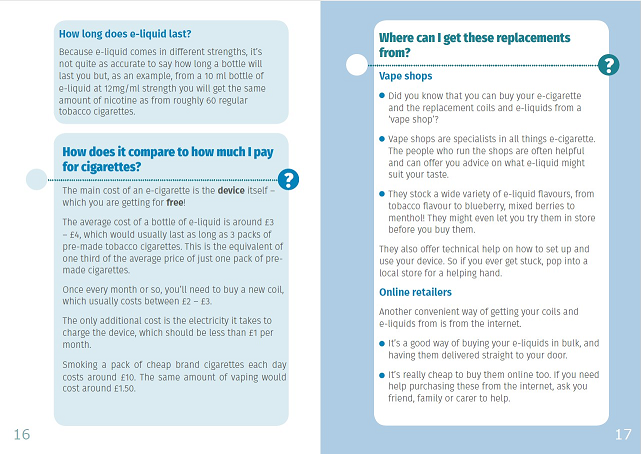
**
